# Multivariate Pattern Analysis of fMRI Reveals Striato‐Cortical Network Changes in Myoclonus‐Dystonia

**DOI:** 10.1111/ene.70085

**Published:** 2025-04-12

**Authors:** Ramesh S. Marapin, Bauke M. de Jong, Remco J. Renken, Elze R. Timmers, Marina A. J. Tijssen, Jelle R. Dalenberg

**Affiliations:** ^1^ Department of Neurology, University Medical Center Groningen University of Groningen Groningen the Netherlands; ^2^ UMCG Expertise Center Movement Disorders Groningen University Medical Center Groningen Groningen the Netherlands; ^3^ Department of Biomedical Sciences of Cells and Systems, University Medical Center Groningen University of Groningen Groningen the Netherlands

**Keywords:** finger tapping, fmri, multivariate analysis, MVPA, myoclonus‐dystonia, searchlight analysis

## Abstract

**Background:**

Currently, the pathophysiology of myoclonus‐dystonia (M‐D) remains insufficiently understood. This study addresses this gap by adding innovative multivariate pattern analysis (MVPA) to traditional univariate analysis of functional magnetic resonance imaging (fMRI) data.

**Methods:**

Data from 18 M‐D patients and 18 age‐matched healthy volunteers who performed a finger tapping fMRI task were analyzed. Whole‐brain univariate and searchlight (MVPA) analysis with varying hemodynamic response function (HRF) delays were employed to examine brain responses associated with the visually guided motor task.

**Results:**

Distinguishing response patterns between M‐D patients and healthy volunteers revealed significant response reductions in the putamen, insula, and visual cortex. Compared to univariate analysis, searchlight analysis was more sensitive for brain activity patterns associated with finger tapping in both M‐D patients and healthy volunteers. At short HRF delays, increased (pre)motor cortical responses were evident in M‐D patients, whereas such responses emerged at a later HRF delay in healthy volunteers.

**Conclusion:**

The task‐related effects observed in M‐D patients support the involvement of the basal ganglia‐thalamo‐cortical network. Notably, cerebellar involvement was not strongly implicated in our study. We postulate that inherent deficits in the putamen trigger either premature or downstream compensatory (motor) cortical effects. The potential involvement of the visual cortex in the M‐D pathophysiology is new, but its role has been suggested by a previous study investigating visual sensory processing in *SGCE* gene‐positive M‐D patients. Our findings, including the innovative searchlight method, pave the way for further studies investigating the complex interplay between brain regions and networks and their role in M‐D pathogenesis.

## 1 Introduction

Myoclonus‐dystonia (M‐D) is a hyperkinetic movement disorder characterized by a combination of myoclonic jerks and dystonia [1]. In about half of cases, M‐D is caused by a mutation in the epsilon‐sarcoglycan (DYT11/*SGCE*) gene [2]. In the remaining cases, the causative genes are often undetermined. The myoclonic jerks are typically the most disabling symptom: while often mild at rest, they become more pronounced during planned movements [3]. Most patients with M‐D also experience dystonia symptoms, which often manifest as cervical dystonia or writer's cramp [4]. In addition to motor symptoms, *SGCE*‐positive M‐D patients have a high incidence of non‐motor symptoms, especially psychiatric, compared to the general population [5, 6, 7, 8, 9]. Roze et al. [3] proposed to limit the term “M‐D phenotype” to patients with an *SGCE‐*like phenotype, including both genetically positive and negative patients to create a phenotypically homogenous group for research purposes. The exact function of *SGCE* in the brain is not fully understood, but it is highly expressed in the Purkinje cells and dentate nucleus of the cerebellum [10].

The origin of the clinical symptoms in M‐D is still undetermined, although several pathophysiological models have been proposed. Electrophysiological studies suggest that the myoclonic jerks have a subcortical origin, based on the absence of cortical hyperexcitability (normal somatosensory evoked potentials and no short‐latency premyoclonic potential on jerk‐locked back averaging) [11, 12, 13, 14]. Subcortical involvement is also hypothesized in the network model for dystonia [15, 16, 17]. M‐D is usually considered a dystonia syndrome with a dysfunctional basal ganglia‐thalamo‐cortical and cerebellothalamocortical network, caused by altered sensorimotor integration, motor inhibition, and sensory processing [3, 18, 19, 20].

It is, however, important to note that not all pathophysiological studies in M‐D are in line with other dystonic syndromes. Electrophysiological studies also show differences between M‐D and other dystonia syndromes, particularly normal intracortical inhibition in M‐D, whereas this is abnormal in primary and focal dystonia [3, 11, 21, 22, 23]. Therefore, brain network functioning remains unclear and warrants further research to inform future diagnostic and therapeutic interventions [19].

Given that planned body movements often exacerbate motor symptoms in M‐D, studying brain function during a motor task using functional magnetic resonance imaging (fMRI) may provide valuable insight into the underlying pathophysiology. To date, only three task‐based fMRI studies have been conducted in M‐D, highlighting aberrant activation in sensorimotor and frontal regions, insula, putamen, and the cerebellum in M‐D patients with *SGCE* mutations [24, 25, 26].

These task‐fMRI studies employed classical univariate (voxel‐wise) analyses to compare the *magnitude* of brain responses between two groups. A relatively new technique, multivariate pattern analysis (MVPA), instead examines *patterns* of neural responses, rather than analyzing single voxel‐ or region‐based values averaged across voxels, as is customary in classical univariate analyses [27, 28]. Searchlight analysis is an MVPA method that can identify informative areas in the brain by using local multivariate patterns. It produces brain response maps by measuring the information in small spherical subsets (“searchlights”) centered on every voxel in the brain [29]. Here, “information” refers to activity patterns in voxels consistently varying with experimental conditions during an fMRI scan (e.g., motor task) [30]. Because MVPA uses patterns of activation across voxels, it is able to detect a broader class of task‐related effects than voxel‐wise analysis, making it often more powerful compared to univariate analysis [31]. Nevertheless, it is recommended to employ both methods as they provide complementary insights into neural mechanisms [32].

In the current study, we employed both traditional univariate analysis and whole‐brain MVPA searchlight analysis to compare neural responses between healthy volunteers and patients with the M‐D phenotype, including both *SGCE* gene‐positive and gene‐negative cases. To this end, we instructed M‐D patients and healthy volunteers to make planned finger‐tapping movements in an MR scanner.

## 2 Materials and Methods

### 2.1 Participants

Nineteen patients with the M‐D clinical phenotype were predominantly recruited from the movement disorder clinic at the UMCG, with a few patients recruited from other hospitals across the Netherlands via their treating physicians. The diagnosis was confirmed by a movement disorders expert (M.T.). Ten patients had a confirmed pathogenic variant in the *SGCE* gene, whereas the other patients did not have this mutation. Nineteen healthy volunteers were selected from the NEMO dataset to match patients on age and gender while prioritizing age over gender.

Participants were included if they were 16 years or older. Exclusion criteria were: (1) other neurological conditions that lead to movement problems other than the hyperkinetic movement disorder, (2) other conditions that lead to impaired hand or arm function, (3) contraindications for MRI. Healthy volunteers who were first‐degree relatives of movement disorder patients were additionally excluded. This study was approved by the medical ethical committee of the UMCG (METc 2018/444). Written informed consent was obtained from all subjects according to the Declaration of Helsinki.

The current study is part of the larger Next Move in Movement Disorders (NEMO) study, a cross‐sectional study at the Expertise Centre Movement Disorders Groningen designed to improve the classification of hyperkinetic movement disorders using machine learning [33].

### 2.2 Clinical Evaluation

Clinical characteristics and demographics of the patients were obtained during study visits; for the complete description of collected clinical information, we refer the reader to the NEMO study protocol paper [33]. M‐D severity was assessed using the clinical global impression severity (CGI‐S) scale, ranging from 1 (normal, no movement disorder) to 7 (among the most extremely affected patients).

### 2.3 MRI Acquisition

Magnetic resonance imaging data were collected on a 3 T Siemens Prisma scanner using a Siemens 64‐channel head coil. T1‐weighted sagittal images (MPRAGE) were acquired at 1 mm isotropic resolution with a repetition time (TR) of 2300 ms and echo time (TE): 2.98 ms. Task‐based fMRI scans were acquired using a multi‐band, multi‐echo (ME) T2*‐weighted echo‐planar sequence with the following scanning parameters: TR = 1.101 ms; TE = 12, 36.1, 60.2 ms; FA = 45°; voxel size = 3.5 mm isotropic, 48 slices, Partial Fourier = 6/8 (no IPAT), MB = 4; bandwidth = 2604 Hz/px; pulse duration = 2560 μs; AP phase encoding direction, MB LeakBlock kernel, for a total duration of 4 min and 46 s. In addition, 10 additional volumes with inverted RO/PE polarity were acquired. The sequence was generously provided by the Center for Magnetic Resonance Research (CMRR) at the University of Minnesota [34].

### 2.4 Task Paradigm

A schematic overview of the fMRI task paradigm is provided in Figure S1. We investigated fMRI‐based brain responses during a bilateral finger tapping task containing 10 trials. During the task, participants received visual cues and instructions in Dutch. The start and duration of every trial were indicated by the text “Right hand” (in Dutch: “Rechter hand,” shown in a red‐colored font) and “Left hand” (“Linker hand,” blue‐colored font) for the right and left finger tapping blocks, respectively. During each block, participants tapped repeatedly and simultaneously with the fingertips of digits two to five on the thumb for 10 s. They were instructed to perform the tapping movement as large and fast as possible. The movement task alternated between the left and the right hand and was repeated five times. All hand‐tapping conditions were separated by a 15‐s rest condition, during which participants were shown a black dot in the center of a gray background. The task lasted for 4 min and 43 s. To ensure correct task performance, we showed an instruction video before the scan and let the participant practice in the scanner before measurements began. In addition, task performance was recorded on video during the scan to monitor task compliance.

### 2.5 Data Preprocessing

MRI data was preprocessed using an in‐house constructed pipeline, which consisted of *fMRIprep* v22.0.2, TE‐dependence analysis (*tedana*) v0.0.12, and Advanced Normalization Tools (*ANTs*) v2.3.5 [35, 36, 37, 38]. For the full data preprocessing pipeline, we refer the reader to the Supporting Information—Data S1.

### 2.6 Univariate Analysis

We first set up a univariate analysis. For each participant, we first smoothed the preprocessed data using an 8 mm FWHM kernel. Subsequently, we set up a mass‐univariate first‐level block design in the statistical package *Nilearn* for Python (v0.9.2, https://nilearn.github.io). As regressors, we included (1) the conditions right hand and left hand, and (2) the realignment parameters, as estimated by fMRIPrep, and their first derivatives as covariates. In addition, a third‐order cosine drift model was used. Data quality was assessed by inspecting the first‐level T‐statistic maps of the right‐ and left‐hand conditions. Patient data sets (and their matched healthy volunteers) that did not show any BOLD signal in the ipsilateral cerebellum and contralateral motor cortex in response to the movement tasks (e.g., due to too severe head movement induced artifacts) were removed from any further group analysis.

Next, we set up a mass univariate group level (i.e., second‐level) analysis using two‐sample T‐tests, to investigate the BOLD contrasts [right hand vs. rest] and [left hand vs. rest] for the M‐D and healthy volunteer groups separately. In addition, we investigated the difference between groups. We considered age and severity as covariates. Statistical tests were performed using two‐sided permutation tests in *Nilearn* (10,000 permutations). For within‐group tests, statistical significance was set at *p* < 0.05 cluster‐wise error correction (with an initial cluster defining threshold of *p* < 0.001). For group comparisons, significance was set more liberally at *p* < 0.001 uncorrected but masked for the [finger tapping vs. rest] cluster‐wise error corrected contrast images to make sure the results coincided with brain areas associated with the task.

### 2.7 Searchlight Multivariate Pattern Analysis

We performed searchlight MVPA analysis using the *BrainIAK* package (v0.11) for Python (Brain Imaging Analysis Kit, http://brainiak.org) [39]. We applied the analysis to distinguish the task conditions [right hand vs. rest] and [left hand vs. rest] separately. We tested searchlights with 5, 7, and 9 mm radii to evaluate their impact on classification accuracy and cluster characteristics (Figure S2). A 7 mm radius was selected as the optimal balance, preserving spatial precision while maximizing accuracy. For the full description of the searchlight multivariate pattern analysis, we refer the reader to the Supporting Information—Data S1.

Subsequently, we performed mass‐univariate one‐sample t‐tests within groups [right hand vs. rest] and [left hand vs. rest], and two‐sample *t*‐tests between groups [right hand patients vs. right hand controls] and [left hand patients vs. left hand controls] on the searchlight maps. Statistical tests were performed using one‐sided and two‐sided permutation tests in *Nilearn* (10,000 permutations), for the within‐group tests and between‐group tests, respectively. We chose to perform one‐sided tests for within‐group tests because negative values in the scaled searchlight maps have no specific meaning (i.e., poor prediction performance). Thresholding was similarly performed as for the group analyses in the univariate analysis described above.

## 3 Results

### 3.1 Clinical Characteristics

An overview of the 18 M‐D patients and 18 matched healthy volunteers that remained in the study is provided in Table 1. An additional 19th participants and a matched healthy volunteer were removed from the analysis due to insufficient data quality; we could not find any BOLD signal response in the cerebellum and motor cortex associated with either left‐ or right‐hand finger tapping. Nine M‐D patients were confirmed carriers of the *SGCE* gene mutation.

**TABLE 1:** Participant characteristics.

M‐D Patients						Matched healthy volunteers	
Age	Gender	R/L	SGCE	Inherited from	CGI‐S	Age	Gender
18	M	R	+	Father	4	20	M
19	M	R	−	−	2.5	23	M
20	M	R	+	Father	2	25	M
20	F	R	−	−	2	20	F
20	M	R	−	−	2	25	M
21	M	R	+	Father	3	28	M
22	M	R	−	−	4	21	F
23	M	R	−	−	3.5	23	F
24	F	R	−	−	3	21	F
27	M	R	+	Father	5	26	F
27	M	R	−	−	3	29	M
39	M	L	+	*De novo*	4	36	M
50	M	R	+	Father	2	50	M
55	M	R	−	−	2	55	M
59	M	R	+	Mother	2	60	M
59	F	L	−	−	4	51	F
63	M	R	+	Father	3.5	60	M
71	M	R	+	Father	4.5	67	M

Abbreviations: CGI‐S, clinical global impression severity; F, female; L, left‐handed; M, male; M‐D, myoclonus‐dystonia; R, right‐handed; SGCE, epsilon‐sarcoglycan.

### 3.2 Univariate fMRI Analysis

To investigate how brain activation differs between healthy volunteers and M‐D patients during finger tapping, we used two‐sample permutation tests on the BOLD contrast maps, separately for the left‐ and right‐hand finger tapping tasks. The resulting contrast images demonstrate which brain areas are hemodynamically more active during the tapping tasks in healthy volunteers compared to M‐D patients.

**FIGURE 1:**
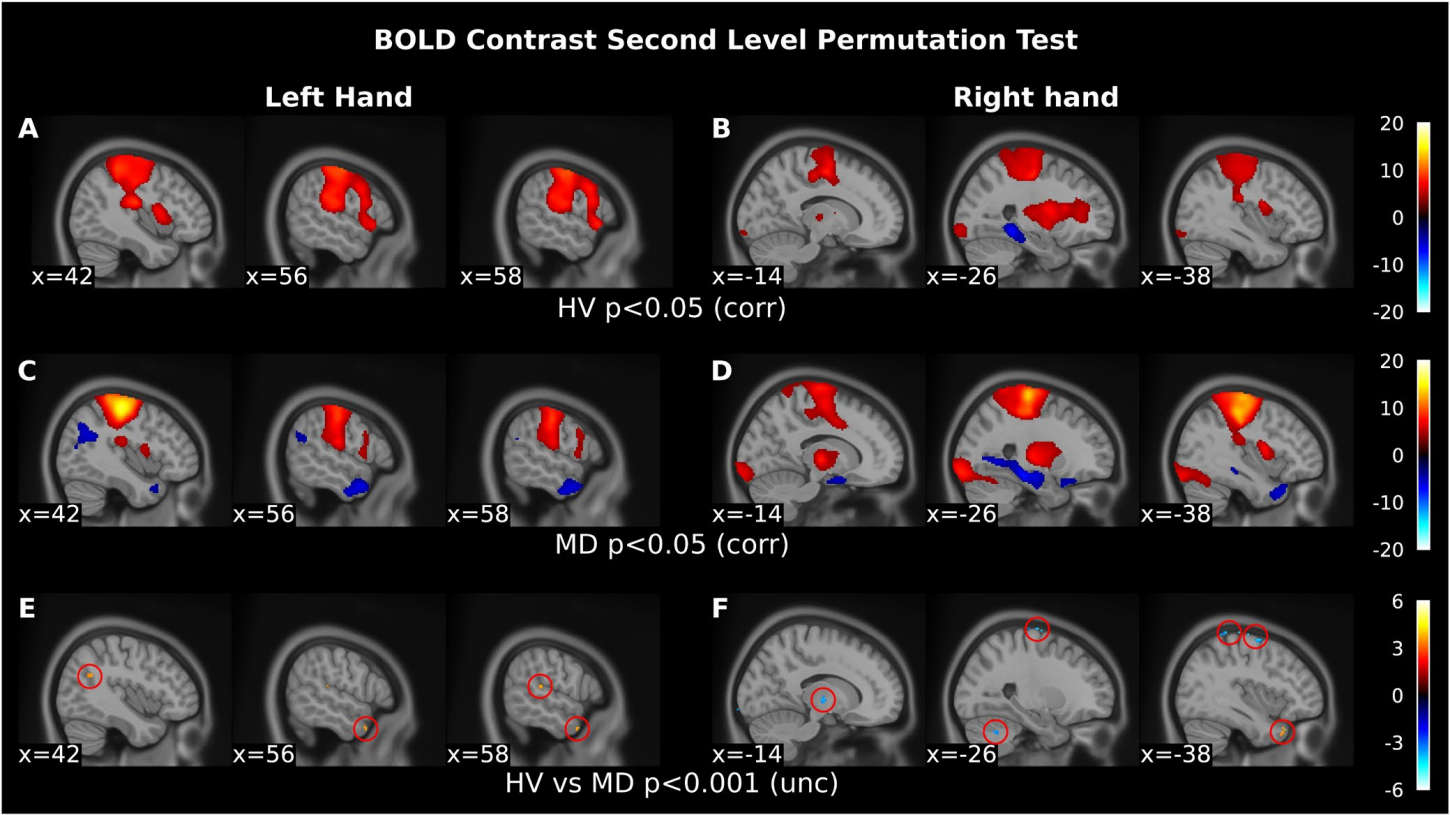
Main BOLD effects for the left‐ and right‐hand finger tapping tasks compared to rest. This figure shows the result of the *Nilearn* mass univariate group analysis. In (A) and (B), these results are shown for the healthy volunteer group, whereas (C) and (D) depict the results for the M‐D group. The result indicates the activated voxels in the motor cortex and cerebellum in response to the finger tapping task compared to the baseline rest condition, which consisted of looking at a white cross on a black screen. (E) and (F) When comparing the contrasts between groups we found decreased activation in the right middle and superior temporal cortex and angular gyrus in M‐D patients during the left finger tapping task. For the right finger tapping task, we found increased activation in the left thalamus, precentral gyrus and cerebellum, and decreased activation in the left superior temporal pole in M‐D patients. BOLD, blood oxygen level‐dependent; HRF, hemodynamic response function; HV, healthy volunteer; M‐D, myoclonus‐dystonia.

Group‐level activation results for the left and right finger tapping tasks compared to rest are shown in Figure 1A–D and Tables S1 and S2, for the M‐D and healthy volunteer groups separately (*p*_FWE_ < 0.05). As expected, we found contralateral activation in the sensorimotor cortex and ipsilateral activation in the cerebellum in response to the tapping tasks, for both groups. When comparing the left‐hand contrast between groups, we found decreased activation in the right middle and superior temporal cortex and angular gyrus in M‐D patients compared to healthy volunteers (*p*_uncorr_ < 0.001; Figure 1E and Table 2). For the right hand, we found increased activation in the left thalamus, precentral gyrus, cerebellum, and decreased activation in the left superior temporal cortex in M‐D patients (*p*_uncorr_ < 0.001; Figure 1F and Table 2).

**TABLE 2:** Main effect of the BOLD contrast (univariate) between‐group analysis for the left and right finger‐tapping task compared to rest.

Cluster	Area	MNI			Peak stat	*p*	*p*	Cluster size
Left hand HV > M‐D		X	Y	Z	(z)	(unc)	(corr)	(mm^3^)
1	Right middle temporal pole	56	12	−30	3.35	< 0.001	ns	216
2	Right superior temporal gyrus	58	‐30	16	3.31	< 0.001	ns	120
3	Right angular gyrus	42	−54	26	3.27	< 0.001	ns	136
Right hand HV > M‐D		X	Y	Z	(z)	(unc)	(corr)	(mm^3^)
1	Left superior temporal pole	−34	22	−30	4.53	< 0.001	ns	216
1	Left precentral gyrus	−36	−6	68	−3.92	< 0.001	ns	184
2	Left precentral gyrus	−26	−12	78	−3.68	< 0.001	ns	200
		−18	−18	80	−3.53	< 0.001		
		−22	−10	78	−3.53	< 0.001		
3	Left cerebellum 6	−28	−58	−36	−3.45	< 0.001	ns	200
4	Left thalamus	−14	−12	0	−3.49	< 0.001	ns	120

Abbreviations: HV, healthy volunteer; M‐D, myoclonus‐dystonia; MNI, Montreal Neurological Institute; ns, non‐significant.

### 3.3 Searchlight MVPA fMRI Analysis

To understand how regional multivariate brain response patterns differ between healthy volunteers and M‐D patients during finger tapping, we used searchlight MVPA to first construct multivariate brain response pattern maps per individual, separately for the left‐ and right‐hand tapping tasks. These response pattern maps indicate which brain areas carry more information about right‐ or left‐hand finger tapping. On a group level, we first investigated these response pattern maps within groups, and then compared the maps between groups to identify regions with the strongest difference in response pattern information between M‐D patients and healthy volunteers.

**FIGURE 2:**
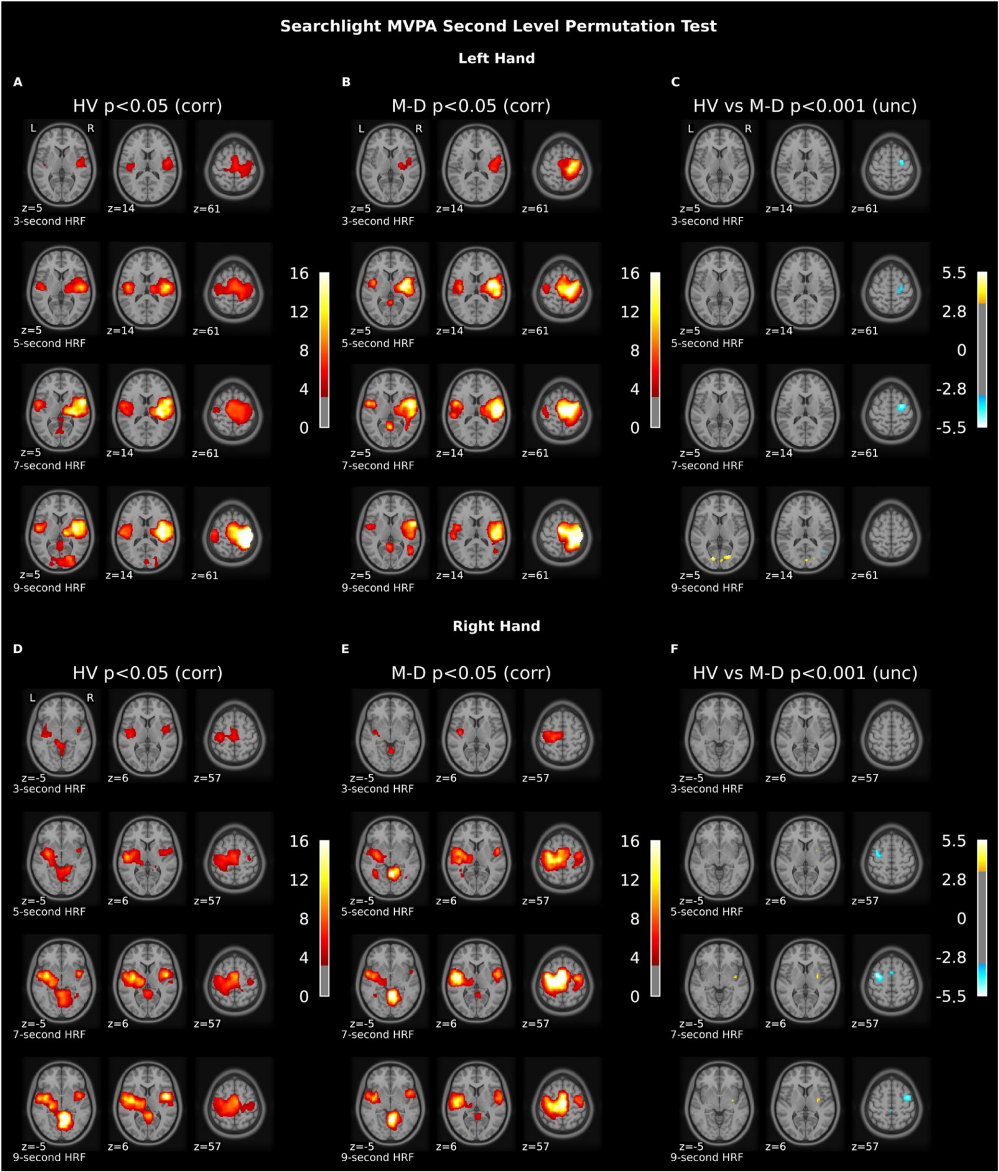
Main searchlight MVPA effects for the left and right finger tapping tasks compared to rest. Panels (A–C) Left hand. Panels (D–F) Right hand. Within a panel, each row represents a different HRF delay (3, 5, 7, and 9 s). This figure shows the result of the searchlight group analysis, specifically, the brain patterns that occur in response to performing the finger tapping tasks. The result indicates the activated voxels in the motor cortex and cerebellum in response to the finger tapping task compared to the baseline rest condition, which consisted of looking at a white cross on a black screen. When comparing the left‐hand contrast between groups (panel C), we found that visual cortex regions contained more regional response pattern information in healthy volunteers compared to patients with M‐D during the late response (from 9 s onwards) (*p*_FWE_ < 0.05). Moreover, we found pattern differences in the thalamus, paracentral lobule, superior frontal, middle cingulate, and lingual gyrus. When comparing the right‐hand contrast between groups (panel F), we found increased putamen and insular response pattern information in healthy volunteers compared to patients with M‐D during the late motor response (from 7 s onwards) (*p*_FWE_ < 0.05) In addition, we found pattern differences in the supplementary motor area, supramarginal, precentral, and postcentral gyrus. HRF, hemodynamic response function; HV, healthy volunteer; M‐D, myoclonus‐dystonia; MVPA, multivariate pattern analysis.

Strikingly, we found a far more extended multivariate response pattern associated with finger tapping than in the univariate analysis (Figure 2 and Tables S3 and S4), indicating that a larger number of brain regions carry information about the task being performed. This response pattern covered cerebellar, motor, visual, and thalamic regions (*p*_FWE_ < 0.05). Furthermore, as the brain response progresses from the early (3‐s HRF delay) to the later (9‐s HRF delay) stage, we observed clear differences in time‐dependent brain responses (Figure 2). In healthy volunteers, the most robust response pattern related to the visually instructed motor task was seen after a 9‐s HRF delay, comprising sensorimotor and occipital cortex activations, together with activations in the cerebellum, putamen, and thalamus (Figure 2). Although bilateral sensorimotor cortex activation was seen, the most pronounced activation was contralateral to the hand movement. This lateralized effect was even stronger for the basal ganglia activations. When, at this late stage, the left‐hand contrast was compared between groups, we found that the bilateral calcarine cortex, left lingual, fusiform, and middle occipital gyrus contained stronger response patterns in healthy volunteers compared to patients with M‐D (*p*_FWE_ < 0.05; Figure 2C). Furthermore, a stronger response in the right thalamus of healthy volunteers was seen at threshold (*p*_uncorr_ < 0.001). At this threshold, cortical responses were reduced in healthy volunteers compared to patients in the right premotor cortex (superior frontal cortex), paracentral lobule, and middle cingulate.

For the right‐hand contrast, after a 7‐s HRF delay, we found that the right putamen and insula contained stronger response patterns in healthy volunteers compared to patients with M‐D (*p*_FWE_ < 0.05), while a reduced cortical response pattern comprised the supplementary motor area, supramarginal, precentral, and postcentral gyrus. (*p*_uncorr_ < 0.001; Figure 2F). In the early stages of the shorter HRF delay, response patterns in sensorimotor cortical regions could already be observed, which were more pronounced in the M‐D patients, identified by significantly reduced responses in healthy volunteers compared to the patient group (Figure 2). Given that task‐related group differences were best captured after 7‐ and 9‐s HRF delays, details of these cluster stats are shown in Table 3.

**TABLE 3:** Main effect of the searchlight between‐group analysis for the left and right finger tapping tasks compared to rest, shown for HRF lag values of 7 and 9 s.

Cluster	Area	MNI			Peak stat	*p*	*p*	Cluster size
		X	Y	Z	(z)	(unc)	(corr)	(mm^3^)
Left hand—HV > M‐D—7 s HRF lag								
1	Right thalamus	6	−24	−6	2.51	< 0.001	ns	936
2	Right lingual gyrus	18	−58	−4	2.17	< 0.001	ns	136
1	Right superior frontal gyrus	26	−4	58	−2.55	< 0.001	ns	5040
2	Right middle cingulate gyrus	4	−32	48	−2.23	< 0.001	ns	480
	Right paracentral lobule	0	−32	58	−2.16	< 0.001	ns	

Left hand—HV > M‐D—9 s HRF lag								
1	Left lingual gyrus	−20	−76	0	2.47	—	< 0.05	2848
	Left fusiform gyrus	−28	−54	−8	2.23	—	< 0.05	
	Left middle occipital gyrus	−32	−80	2	2.13	—	< 0.05	
2	Right calcarine gyrus	22	−76	8	2.43	—	< 0.05	2304
	Left calcarine gyrus	2	−86	10	2.28	—	< 0.05	
3	Right thalamus	12	−20	−4	2.30	< 0.001	ns	376

Right hand—HV > M‐D—7 s HRF lag								
1	Right putamen	32	−6	2	2.30	—	< 0.05	2232
	Right insula	40	−8	−6	2.21	—	< 0.05	
1	Left precentral gyrus	−34	−2	60	−2.67	< 0.001	ns	9000
	Right supplementary motor area	8	−6	70	−2.35	< 0.001	ns	
	Left supplementary motor area	−4	4	62	−2.24	< 0.001	ns	
2	Right precentral gyrus	46	6	48	−2.30	< 0.001	ns	664
	Right postcentral gyrus	50	−22	46	−2.19	< 0.001	ns	104
3	Left postcentral gyrus	−36	−34	48	−2.13	< 0.001	ns	152

Right hand—HV > M‐D—9 s HRF lag								
1	Right putamen	32	−8	2	2.32	—	< 0.05	1080
2	Left thalamus	−22	−26	0	2.16	< 0.001	ns	80
1	Right middle frontal gyrus	44	0	54	−2.48	< 0.001	ns	3408
2	Right supplementary motor area	4	0	70	−2.33	< 0.001	ns	2728
	Right supplementary motor area	10	−14	68	−2.18	< 0.001	ns	
3	Paracentral lobule	0	−32	60	−2.16	< 0.001	ns	136

Abbreviations: HRF, hemodynamic response function; HV, healthy volunteer; M‐D, myoclonus‐dystonia; MNI, Montreal Neurological Institute; ns, non‐significant.

## 4 Discussion

In this study, we examined altered neural activity in patients with the M‐D phenotype (both with and without an *SGCE* mutation) using functional MRI during a bilateral finger tapping task. We employed traditional univariate and whole‐brain MVPA searchlight analysis to compare neural responses between M‐D patients and healthy volunteers. The results of the searchlight analysis were more robust, with more widespread brain activity patterns associated with visually instructed finger tapping in both M‐D patients and healthy volunteers compared to univariate analysis. Moreover, while both methods identified group differences with a liberal threshold, only searchlight analysis found significant differences. This observation underscores the superior performance of MVPA compared to univariate methods and supports its effectiveness in distinguishing between patient and healthy populations [40, 41, 42, 43, 44, 45]. The response patterns that significantly differed between M‐D patients and healthy volunteers comprised the basal ganglia, insula, and visual cortex. Notably, (pre)motor cortical differences appeared earlier in patients (short HRF delay) than the pronounced task‐related response in healthy volunteers.

For M‐D patients, enhanced BOLD response patterns were found in the precentral gyrus and supplementary motor areas during short HRF delays (3–5 s post stimulus), which persisted for the lateral premotor cortex at longer HRF delays. These results were similar across the left and right hand finger tapping tasks. During late HRF delays (7–9 s), enhanced BOLD response patterns in cortical motor regions co‐occurred with reduced response patterns in the right putamen and insula during right‐hand tapping. These results align with prior task‐fMRI findings in patients with M‐D, where similar regions are implicated [25, 26]. Furthermore, the involvement of these brain regions has also been reported in previous neuroimaging studies in patients with focal dystonia [46, 47, 48, 49, 50, 51]. As normal task performance involves concurrent BOLD increases in the (pre)motor cortex and striatum, the opposite pattern in these regions suggests a pathophysiological mechanism where impaired striatum function triggers network effects on cortical motor regions, either as premature activation at task onset [52] or downstream compensation. This aligns with M‐D literature indicating primary basal ganglia network involvement, as cortical physiological measurements did not show major disturbances [4].

During late HRF delays, reduced BOLD response patterns were observed in the visual cortex of patients compared to healthy volunteers during left‐hand finger tapping, indicating potential occipital region alterations. This aligns with the recent study by Tarrano et al. [53] who reported visual temporal discrimination impairments linked to visual cortex volume changes in SGCE‐positive M‐D. The visual cortex plays a crucial role in processing visual information and transmitting it to various neural structures involved in perception [54]. In our study, participants performed a finger‐tapping task while reading visually presented instructions, highlighting the role of the visual cortex in the task at hand, and thus potentially serving visuomotor binding. As ventral (as opposed to dorsal) visual regions were particularly implicated in task performance by healthy volunteers, such binding likely pertained more to the textual aspects of the task rather than visuospatial function. These alterations may also relate to impaired reading and ascribing meaning to words rather than directly impacting motor performance. In their study, Tarrano et al. [53] argued that altered function of the visual saliency map may indicate abnormal visuomotor transformation in M‐D patients. In this respect, the visual cortex is considered to participate in a network also comprising the superior colliculus, putamen, and thalamus, underpinning temporal discrimination [55], that is, the ability to perceive two sequential sensory stimuli separate in time. Altogether, the results described by Tarrano et al. [53] and our present study point to an important role of the visual cortex in M‐D patients, which is not limited to impaired visual saliency maps. To gain a more comprehensive understanding of these mechanisms, future studies should further explore visual function and its correlation with the motor phenotype in M‐D patients. This may contribute valuable insights toward elucidating the interplay between visual processing and motor manifestations in M‐D.

Although cerebellar activation was obvious during the task performance, we did not identify patient‐related changes in this region, despite its recognized importance in the motor network affected in M‐D [3]. One explanation could be that the cerebellum may function effectively locally but face communication (connectivity) challenges within the broader motor control network, especially with the thalamus and basal ganglia, which was not assessed by our current methodology, as we did not investigate functional connectivity. On the other hand, the reduced response pattern in the thalamus in patients compared to healthy volunteers, as identified by searchlight analysis, might hint at a cerebellar output effect as the thalamus is a major output target of the cerebellum, as well as for the basal ganglia. This finding aligns with the current perspective on dystonia pathophysiology, where recent research suggests that aberrant inputs from the cerebellum to the basal ganglia may underlie the condition, without necessarily implicating the cerebellum as the primary or sole neuroanatomical site of origin [56]. In this context, there may be a key role for the cerebello‐thalamo‐striatal pathway in M‐D and dystonia, wherein cerebellar dysfunction could contribute to dystonia by transmitting aberrant input to the striatum, leading to abnormal control of neural plasticity [17]. Subsequent studies investigating functional connectivity in M‐D patients can help shed light on this mechanism. Finally, it is important to consider that the cohort examined in this study included both *SGCE* gene‐positive and *SGCE* gene‐negative M‐D patients. While the primary focus of this study was to delve into the mechanisms of the M‐D phenotype, future studies should explore whether distinct M‐D subgroups, categorized based on their genetic (*SGCE*) status, exhibit variations in their pathophysiology.

## 5 Conclusion

The task‐related effects revealed in (pre)motor cortical regions and putamen of M‐D patients provide further specification of the involvement of basal ganglia‐thalamo‐cortical network components in M‐D. We postulate that inherent deficits in the putamen induce either premature or downstream compensatory cortical effects. Furthermore, our findings also lend support to the putative involvement of the visual cortex as a novel brain region in the pathophysiology of M‐D. Notably, our study did not strongly implicate the cerebellum. The task‐evoked fMRI measurements of the present study enabled the identification of distinct functional network changes in M‐D. These findings may guide the design of further research to explore which constituents of these networks are pathologically affected at the tissue level, and what mechanism may lead to distributed network effects underlying the manifestation of symptoms, thus further elucidating the complex interplay between the brain regions identified in our study and their role in the disorder's pathogenesis.

## Author Contributions

**Ramesh S. Marapin:** conceptualization, funding acquisition, investigation, writing – original draft, methodology, writing – review and editing, visualization, formal analysis, project administration, software. **Bauke M. de Jong:** writing – original draft, conceptualization, writing – review and editing, formal analysis, supervision, investigation, methodology. **Remco J. Renken:** writing – review and editing, supervision. **Elze R. Timmers:** investigation, writing – review and editing, supervision. **Marina A. J. Tijssen:** resources, supervision, writing – review and editing, investigation, conceptualization, funding acquisition. **Jelle R. Dalenberg:** data curation, supervision, project administration, formal analysis, software, methodology, validation, conceptualization, investigation.

## Conflicts of Interest

The authors declare no conflicts of interest.

## Supporting information


Data S1.

## Data Availability

The data that support the findings of this study are available on request from the corresponding author. The data are not publicly available due to privacy or ethical restrictions.
