# Supplementary material for: Multivariate Pattern Analysis of fMRI Reveals Striato‐Cortical Network Changes in Myoclonus‐Dystonia
Source: Eur J Neurol. 2025 Apr 12;32(4):e70085. doi: 10.1111/ene.70085 (PMC11992478; doi:10.1111/ene.70085)
Supplement: Supplementary file 1 — Data S1. [file ENE-32-e70085-s001.docx]

# Supplementary Material

## Data preprocessing

First, *fMRIprep* preprocessing was applied to the data using the “single-echo” output parameter. Subsequently, minimally preprocessed single echo time course data were submitted to multi-echo independent component analysis (*ME-ICA*) denoising using the Python library *tedana*. In this step, we used an individual-based whole brain mask that was calculated based on the blood oxygen level-dependent (BOLD)-reference file generated by *fMRIPrep*. The resulting denoised BOLD time series was normalized to MNI space with *ANTs* using the transformation file generated by *fMRIPrep*, and finally resliced to 2 mm isotropic voxels. The resulting preprocessed files were used as input for a classical univariate analysis, and a searchlight multivariate pattern analysis.

## Searchlight MVPA fMRI analysis

For each searchlight, we used a cube shape with a radius of 7 mm. Furthermore, to evaluate early and late brain response patterns during motor responses, we examine response patterns using hemodynamic response function (HRF) delay values of 3,5, 7, and 9 seconds. For each sphere, a radial basis function (RBF) support vector machine (SVM) was trained and validated in a stratified group 5-fold cross-validation framework, provided in the *scikit-learn* (v1.1.0) package for Python. In this framework, timepoints belonging to each block condition were grouped together, and for each cross-validation fold, four movement and rest conditions were used as training while one movement and rest condition were used as validation. Model performance was expressed as the average area under the curve (AUC) of the receiver operating characteristic (ROC) curve across folds. The average AUC scores were then projected to the centre voxel of the searchlight.

Since we observed variable levels of bias across all participants in the histograms of the AUC searchlight maps, we first scaled each map using a robust scaling method by removing the median and scaling the data to the inter-quartile range.

**Supplementary Table 1**

**Main effect of the BOLD contrast (univariate) within-group analysis** **for the left finger tapping task compared to rest (P_(FWE)_ < 0.05).**

| **Cluster** | **Area** | **MNI** | | | **Peak stat** | **Cluster Size** |
| --- | --- | --- | --- | --- | --- | --- |
|  | | **X** | **Y** | **Z** | **(z)** | **(mm3)** |
| **HV** | |  |  |  |  |  |
| 1 | Right postcentral gyrus | 28 | -46 | 70 | 5.67 | 157840 |
|  | Right thalamus | 20 | -16 | 6 | 5.57 |  |
|  | Right superior parietal gyrus | 20 | -54 | 60 | 5.51 |  |
|  | Right inferior parietal gyrus | 56 | -14 | 56 | 5.49 |  |
| 2 | Left cerebellum 4+5 | -4 | -54 | -14 | 5.03 | 32496 |
|  | Left cerebellum 8 | -18 | -56 | -52 | 4.91 |  |
|  | Left cerebellum 4+5 | -22 | -38 | -24 | 4.79 |  |
|  | Left cerebellum 8 | -30 | -58 | -56 | 4.78 |  |
| 3 | Left supramarginal gyrus | -66 | -18 | 42 | 5.01 | 25360 |
|  | Left supramarginal gyrus | -48 | -30 | 20 | 4.78 |  |
|  | Left supramarginal gyrus | -58 | -20 | 20 | 4.68 |  |
|  | Left precentral gyrus | -64 | 2 | 34 | 4.62 |  |
| 4 | Left insula | -42 | 0 | 12 | 4.60 | 9336 |
|  | Left putamen | -20 | 2 | 14 | 3.99 |  |
|  |  |  |  |  |  |  |
| 1 | Left cuneus | -10 | -88 | 34 | -3.89 | 4144 |
|  | Left cuneus | -14 | -94 | 24 | -3.75 |  |
|  | Left superior occipital gyrus | -12 | -104 | 18 | -3.55 |  |
| **M-D** | |  |  |  |  |  |
| 1 | Right precentral gyrus | 40 | -22 | 58 | 6.51 | 100840 |
|  | Right supplementary motor area | 4 | -4 | 54 | 5.26 |  |
|  | Right supramarginal gyrus | 60 | -18 | 44 | 5.22 |  |
|  | Right supplementary motor area | 6 | -8 | 78 | 5.16 |  |
| 2 | Left cerebellum 4+5 | -16 | -52 | -20 | 5.69 | 41840 |
|  | Left cerebellum 8 | -20 | -58 | -52 | 5.41 |  |
|  | Vermis 6 | -2 | -58 | -20 | 5.29 |  |
|  | Left inferior occipital gyrus | -20 | -100 | -12 | 4.69 |  |
| 3 | Left inferior parietal lobe | -56 | -26 | 46 | 4.90 | 18488 |
|  | Left supramarginal gyrus | -58 | -22 | 24 | 4.43 |  |
|  | Left supramarginal gyrus | -48 | -36 | 26 | 4.06 |  |
|  | Left superior parietal lobe | -36 | -46 | 70 | 3.43 |  |
| 4 | Right thalamus | 16 | -14 | 2 | 4.17 | 10176 |
|  | Right thalamus | 18 | -16 | 10 | 4.13 |  |
|  | Right pallidum | 18 | -4 | -6 | 3.99 |  |
|  | Right putamen | 30 | -10 | 2 | 3.89 |  |
| 5 | Left Rolandic operculum | -46 | 0 | 12 | 4.56 | 5776 |
|  | Left precentral gyrus | -60 | 4 | 36 | 4.02 |  |
| 6 | Right Insula | 36 | 4 | 14 | 4.14 | 5080 |
|  | Right inferior frontal gyrus, opercular | 62 | 14 | 30 | 3.94 |  |
|  | Right inferior frontal gyrus, opercular | 56 | 8 | 10 | 3.80 |  |
|  | Right Rolandic operculum | 64 | 12 | 6 | 3.67 |  |
| 7 | Left precentral gyrus | -38 | -10 | 64 | 4.64 | 4448 |
|  |  |  |  |  |  |  |
| 1 | Left precuneus | -6 | -48 | 36 | 4.17 | 14760 |
|  | Right precuneus | 10 | -58 | 28 | 4.17 |  |
|  | Right middle cingulate gyrus | 6 | -42 | 32 | -3.84 |  |
| 2 | Left angular gyrus | -56 | -64 | 26 | -5.08 | 11296 |
|  | Left angular gyrus | -50 | -58 | 36 | -4.31 |  |
|  | Left middle occipital gyrus | -34 | -90 | 32 | -3.49 |  |
|  | Left superior occipital gyrus | -24 | -84 | 26 | -3.28 |  |
| 3 | Right angular gyrus | 46 | -60 | 28 | -4.24 | 15448 |
|  | Right superior occipital gyrus | 16 | -90 | 18 | -4.05 |  |
|  | Left cuneus | -8 | -92 | 18 | -3.77 |  |
|  | Right superior occipital gyrus | 18 | -90 | 32 | -3.51 |  |
| 4 | Right middle temporal gyrus | 52 | 8 | -28 | -4.72 | 5672 |
|  | Right inferior temporal gyrus | 58 | -6 | -34 | -4.16 |  |
| 5 | Left middle temporal gyrus | -58 | -8 | -10 | -4.10 | 2800 |
|  | Left middle temporal gyrus | -62 | -20 | -6 | -3.61 |  |

**Supplementary Table 2**

**Main effect of the BOLD contrast (univariate) within-group analysis** **for the right finger tapping task compared to rest (P_(FWE)_ < 0.05).**

| **Cluster** | **Area** | **MNI** | | | **Peak stat** | **Cluster Size** |
| --- | --- | --- | --- | --- | --- | --- |
|  | | **X** | **Y** | **Z** | **(z)** | **(mm3)** |
| **HV** | |  |  |  |  |  |
| 1 | Left precentral gyrus | -22 | -18 | 52 | 5.32 | 80064 |
|  | Left precentral gyrus | -18 | -10 | 54 | 5.26 |  |
|  | Left precentral gyrus | -62 | 8 | 28 | 4.86 |  |
|  | Right supplementary motor area | 14 | 2 | 56 | 4.63 |  |
| 2 | Left putamen | -24 | 2 | 12 | 4.91 | 25264 |
|  | Left insula | -34 | 0 | 18 | 4.65 |  |
|  | Left putamen | -28 | -2 | -2 | 4.46 |  |
|  | Left insula | -26 | 18 | 16 | 4.46 |  |
| 3 | Right postcentral gyrus | 64 | -14 | 30 | 4.71 | 12864 |
|  | Right inferior parietal lobule | 38 | -42 | 48 | 3.64 |  |
| 4 | Right Rolandic operculum | 46 | 4 | 16 | 4.63 | 18008 |
|  | Right precentral gyrus | 58 | 10 | 20 | 4.26 |  |
|  | Right insula | 26 | 32 | 10 | 4.12 |  |
|  | Right putamen | 24 | 16 | 8 | 3.87 |  |
| 5 | Right inferior occipital gyrus | 30 | -96 | -6 | 4.35 | 3376 |
| 6 | Right cerebellum 4+5 | 16 | -50 | -20 | 4.20 | 16928 |
|  | Right cerebellum 8 | 10 | -66 | -38 | 4.00 |  |
|  | Right cerebellum 8 | 16 | -66 | -44 | 3.95 |  |
|  | Right cerebellum 8 | 20 | -54 | -54 | 3.93 |  |
| 7 | Left inferior occipital gyrus | -28 | -96 | -8 | 4.11 | 3936 |
|  |  |  |  |  |  |  |
| 1 | Left fusiform gyrus | -26 | -40 | -10 | -4.89 | 3248 |
| **M-D** | |  |  |  |  |  |
| 1 | Left precentral gyrus | -28 | -22 | 72 | 6.17 | 108344 |
|  | Left postcentral gyrus | -38 | -28 | 54 | 6.09 |  |
|  | Left precentral gyrus | -40 | -20 | 68 | 6.00 |  |
|  | Right supplementary motor area | 8 | -2 | 58 | 5.34 |  |
| 2 | Right cerebellum 4+5 | 18 | -50 | -22 | 6.02 | 54056 |
|  | Right cerebellum 8 | 14 | -66 | -54 | 5.33 |  |
|  | Left lingual gyrus | -30 | -88 | -14 | 5.04 |  |
|  | Left cerebellum 6 | -32 | -68 | -18 | 4.37 |  |
| 3 | Left thalamus | -18 | -16 | 2 | 5.25 | 21880 |
|  | Left insula | -42 | 2 | 12 | 4.99 |  |
| 4 | Right calcarine | 22 | -98 | -4 | 5.12 | 11040 |
| 5 | Right supramarginal gyrus | 56 | -30 | 28 | 4.21 | 14808 |
|  | Right supramarginal gyrus | 48 | -28 | 38 | 4.14 |  |
|  | Right Rolandic operculum | 54 | -18 | 24 | 4.02 |  |
| 6 | Right Rolandic operculum | 50 | 8 | 6 | 4.16 | 4472 |
|  | Right insula | 36 | 20 | 10 | 3.36 |  |
|  |  |  |  |  |  |  |
| 1 | Left parahippocampal gyrus | -30 | -30 | -12 | -4.88 | 22992 |
|  | Left lingual gyrus | -28 | -60 | 0 | -4.49 |  |
|  | Left inferior temporal gyrus | -56 | -16 | -24 | -4.38 |  |
|  | Left superior temporal pole | -34 | 22 | -28 | -4.28 |  |
| 2 | Right rectal gyrus | 8 | 32 | -18 | -4.51 | 4016 |
|  | Right rectal gyrus | 2 | 24 | -16 | -4.35 |  |
| 3 | Right middle temporal gyrus | 58 | -2 | -28 | -4.08 | 6952 |
|  | Right middle temporal pole | 36 | 20 | -36 | -4.02 |  |
|  | Right middle temporal pole | 56 | 16 | -28 | -3.92 |  |

**Supplementary Table 3. Main effect of the searchlight analysis** **for the left finger tapping task compared to rest for different HRF delays (3, 5, 7, and 9 seconds) (within-group P_(FWE)_ < 0.05; between-group P < 0.001).**

| **Cluster** | **Area** | **MNI** | | | **Peak stat** | **Cluster Size** |
| --- | --- | --- | --- | --- | --- | --- |
|  | | **X** | **Y** | **Z** | **(z)** | **(mm3)** |
| **HV – 3 second HRF lag** | |  |  |  |  |  |
| 1 | Right middle cingulate gyrus | 14 | -14 | 48 | 2.56 | 75752 |
|  | Right supplementary motor area | 10 | -22 | 54 | 2.48 |  |
|  | Right supplementary motor area | 10 | -4 | 54 | 2.42 |  |
|  | Right precentral gyrus | 40 | -14 | 46 | 2.37 |  |
| 2 | Vermis 4+5 | -4 | -56 | -24 | 2.38 | 24848 |
|  | Left cerebellum 6 | -8 | -62 | -14 | 2.33 |  |
|  | Left cerebellum 4+5 | -16 | -40 | -24 | 2.07 |  |
|  | Left cerebellum 4+5 | -26 | -42 | -22 | 2.03 |  |
| 3 | Left Rolandic operculum | -46 | -22 | 20 | 2.36 | 5496 |
|  | Left superior temporal gyrus | -44 | -36 | 20 | 2.02 |  |
|  | Left insula | -36 | -16 | 14 | 1.99 |  |
| **MD – 3 second HRF lag** | |  |  |  |  |  |
| 1 | Left cerebellum 4+5 | -10 | -40 | -12 | 3.08 | 40344 |
|  | Left cerebellum 4+5 | -22 | -44 | -24 | 2.54 |  |
|  | Vermis 8 | 0 | -60 | -30 | 2.26 |  |
|  | Right cerebellum 4+5 | 24 | -42 | -26 | 2.10 |  |
| 2 | Right postcentral gyrus | 30 | -24 | 46 | 3.06 | 113656 |
|  | Right postcentral gyrus | 48 | -20 | 38 | 3.02 |  |
|  | Right postcentral gyrus | 22 | -26 | 58 | 2.93 |  |
|  | Right superior frontal gyrus | 30 | -12 | 62 | 2.88 |  |
| **HV > MD – 3 second HRF lag** | |  |  |  |  |  |
| 1 | Right superior frontal gyrus | 28 | -6 | 64 | -2.38 | 1064 |
| 2 | Right postcentral gyrus | 22 | -38 | 52 | -2.33 | 2688 |
|  | Right inferior parietal lobule | 24 | -48 | 52 | -2.32 |  |
|  | Right postcentral gyrus | 28 | -22 | 48 | -2.26 |  |
|  | Right paracentral lobule | 14 | -32 | 52 | -2.23 |  |
| **HV – 5 second HRF lag** | |  |  |  |  |  |
| 1 | Left fusiform gyrus | -32 | -58 | -16 | 2.89 | 77224 |
|  | Vermis 6 | 0 | -62 | -18 | 2.87 |  |
|  | Left fusiform gyrus | -32 | -48 | -18 | 2.85 |  |
|  | Left cerebellum 6 | -22 | -60 | -12 | 2.76 |  |
| 2 | Right Rolandic operculum | 50 | -10 | 14 | 2.80 | 171136 |
|  | Right supplementary motor area | 8 | -4 | 50 | 2.72 |  |
|  | Right middle cingulate gyrus | 12 | -14 | 50 | 2.72 |  |
|  | Left middle cingulate gyrus | -4 | -4 | 48 | 2.68 |  |
| 3 | Left Rolandic operculum | -46 | -12 | 14 | 2.61 | 17480 |
| **MD – 5 second HRF lag** | |  |  |  |  |  |
| 1 | Left precuneus | -16 | -50 | -18 | 3.32 | 96424 |
|  | Left cerebellum 4+5 | -24 | -44 | -26 | 3.19 |  |
|  | Vermis 4+5 | -2 | -52 | -4 | 2.98 |  |
|  | Vermis 6 | 2 | -66 | -22 | 2.91 |  |
| 2 | Right precentral gyrus | 40 | -14 | 46 | 3.22 | 239816 |
|  | Right postcentral gyrus | 34 | -26 | 46 | 3.18 |  |
|  | Right postcentral gyrus | 52 | -24 | 42 | 3.17 |  |
|  | Right middle cingulate gyrus | 20 | -24 | 48 | 3.13 |  |
| **HV > MD – 5 second HRF lag** | |  |  |  |  |  |
| 1 | Right postcentral gyrus | 28 | -38 | 50 | -2.51 | 9576 |
|  | Right superior frontal gyrus | 28 | -10 | 58 | -2.19 |  |
|  | Right supramarginal gyrus | 56 | -26 | 38 | -2.17 |  |
|  | Right supramarginal gyrus | 48 | -32 | 40 | -2.17 |  |
| 2 | Left cerebellum 8 | -28 | -38 | -44 | -2.15 | 80 |
| **HV – 7 second HRF lag** |  |  |  |  |  |  |
| 1 | Right middle cingulate gyrus | 12 | -12 | 46 | 3.13 | 317184 |
|  | Right Rolandic operculum | 52 | -18 | 24 | 3.01 |  |
|  | Right Heschl’s gyrus | 36 | -24 | 12 | 3.01 |  |
|  | Right Rolandic operculum | 52 | -22 | 22 | 3.00 |  |
| 2 | Left Rolandic operculum | -44 | -26 | 24 | 2.63 | 39952 |
|  | Left Rolandic operculum | -54 | -2 | 10 | 2.54 |  |
|  | Left insula | -36 | -12 | 16 | 2.24 |  |
|  | Left precentral gyrus | -42 | -10 | 52 | 2.17 |  |
| **MD – 7 second HRF lag** |  |  |  |  |  |  |
| 1 | Right middle cingulate gyrus | 2 | -26 | 46 | 3.41 | 337896 |
|  | Right precentral gyrus | 38 | -2 | 52 | 3.22 |  |
|  | Right middle cingulate gyrus | 2 | -10 | 42 | 3.19 |  |
|  | Right postcentral gyrus | 46 | -22 | 40 | 3.17 |  |
| 2 | Left supramarginal gyrus | -52 | -26 | 22 | 2.94 | 48880 |
|  | Left Rolandic operculum | -50 | 2 | 12 | 2.87 |  |
|  | Left inferior parietal lobule | -56 | -22 | 40 | 2.38 |  |
|  | Left postcentral gyrus | -44 | -32 | 50 | 2.36 |  |
| **HV > MD – 7 second HRF lag** |  |  |  |  |  |  |
| 1 | Right thalamus | 6 | -24 | -6 | 2.51 | 936 |
| 2 | Right lingual gyrus | 18 | -58 | -4 | 2.17 | 136 |
|  |  |  |  |  |  |  |
| 1 | Right superior frontal gyrus | 26 | -4 | 58 | -2.55 | 5040 |
| 2 | Right middle cingulate gyrus | 4 | -32 | 48 | -2.23 | 480 |
|  | Right paracentral lobule | 0 | -32 | 58 | -2.16 |  |
| **HV – 9 second HRF lag** | |  |  |  |  |  |
| 1 | Right postcentral gyrus | 32 | -42 | 60 | 3.13 | 214984 |
|  | Right postcentral gyrus | 32 | -28 | 58 | 3.10 |  |
|  | Right postcentral gyrus | 40 | -32 | 52 | 3.08 |  |
|  | Right supramarginal gyrus | 50 | -26 | 40 | 3.07 |  |
| 2 | Left lingual gyrus | -24 | -50 | -10 | 3.13 | 168840 |
|  | Vermis 4+5 | 0 | -58 | -10 | 3.07 |  |
|  | Left cerebellum 6 | -14 | -52 | -26 | 3.04 |  |
|  | Left cerebellum 6 | -26 | -56 | -26 | 2.97 |  |
| 3 | Left inferior parietal lobule | -54 | -24 | 46 | 2.65 | 46968 |
|  | Left superior temporal gyrus | -52 | -8 | 6 | 2.64 |  |
|  | Left supramarginal gyrus | -56 | -20 | 20 | 2.57 |  |
|  | Left supramarginal gyrus | -48 | -22 | 26 | 2.55 |  |
| **MD – 9 second HRF lag** | |  |  |  |  |  |
| 1 | Right precentral gyrus | 18 | -20 | 70 | 3.21 | 199264 |
|  | Left supplementary motor area | -4 | -8 | 60 | 3.16 |  |
|  | Right postcentral gyrus | 54 | -18 | 48 | 3.15 |  |
|  | Right postcentral gyrus | 24 | -44 | 62 | 3.01 |  |
| 2 | Left cerebellum 4+5 | -4 | -48 | -2 | 3.19 | 105512 |
|  | Right cerebellum 4+5 | 8 | -56 | -10 | 3.18 |  |
|  | Left fusiform gyrus | -20 | -44 | -10 | 3.16 |  |
|  | Left cerebellum 6 | -4 | -70 | -18 | 3.03 |  |
| 3 | Left superior temporal gyrus | -54 | -26 | 18 | 2.60 | 14168 |
|  | Left Rolandic operculum | -48 | -2 | 12 | 2.52 |  |
| **HV > MD – 9 second HRF lag** |  |  |  |  |  |  |
| 1 | Left lingual gyrus | -20 | -76 | 0 | 2.47 | 1792 |
|  | Left occipital middle gyrus | -32 | -80 | 2 | 2.13 |  |
| 2 | Right calcarine | 22 | -76 | 8 | 2.43 | 976 |
| 3 | Right thalamus | 12 | -20 | -4 | 2.30 | 376 |
| 4 | Left calcarine | 2 | -86 | 10 | 2.28 | 616 |
| 5 | Left fusiform gyrus | -28 | -54 | -8 | 2.23 | 128 |

**Supplementary Table 4. Main effect of the searchlight analysis** **for the right finger tapping task compared to rest for different HRF delays (3, 5, 7, and 9 seconds) (within-group P_(FWE)_ < 0.05; between-group P < 0.001).**

| **Cluster** | **Area** | **MNI** | | | **Peak stat** | **Cluster Size** |
| --- | --- | --- | --- | --- | --- | --- |
|  | | **X** | **Y** | **Z** | **(z)** | **(mm3)** |
| **HV – 3 second HRF lag** | |  |  |  |  |  |
| 1 | Vermis 4+5 | 4 | -54 | -18 | 2.44 | 15048 |
|  | Left cerebellum 4+5 | -6 | -38 | -2 | 2.30 |  |
|  | Right cerebellum 6 | 16 | -60 | -22 | 2.07 |  |
| 2 | Left Heschl’s gyrus | -42 | -14 | 10 | 2.36 | 16904 |
|  | Left superior temporal gyrus | -46 | -20 | 0 | 2.23 |  |
|  | Left Rolandic operculum | -50 | -6 | 8 | 2.23 |  |
|  | Left putamen | -30 | -16 | -2 | 2.21 |  |
| 3 | Left paracentral lobule | 0 | -22 | 54 | 2.35 | 49208 |
|  | Right supplementary motor area | 8 | -6 | 52 | 2.28 |  |
|  | Left postcentral gyrus | -38 | -20 | 54 | 2.28 |  |
|  | Left supplementary motor area | 0 | -8 | 68 | 2.27 |  |
| **MD – 3 second HRF lag** | |  |  |  |  |  |
| 1 | Right cerebellum 3 | 14 | -40 | -30 | 2.66 | 16432 |
|  | Vermis 8 | 8 | -56 | -26 | 2.35 |  |
|  | Vermis 4+5 | 2 | -56 | -6 | 2.33 |  |
|  | Right cerebellum 4+5 | 28 | -36 | -28 | 2.19 |  |
| 2 | Right insula | -40 | -12 | 4 | 2.44 | 6280 |
|  | Left putamen | -32 | -12 | -8 | 2.14 |  |
| 3 | Left precentral gyrus | -22 | -26 | 58 | 2.43 | 29832 |
|  | Left paracentral lobule | -8 | -18 | 56 | 2.40 |  |
|  | Left precentral gyrus | -34 | -14 | 56 | 2.33 |  |
|  | Left middle cingulate gyrus | -6 | -2 | 48 | 2.19 |  |
| 4 | Right supplementary motor area | 10 | -12 | 74 | 2.12 | 3664 |
|  | Left supplementary motor area | -6 | -4 | 76 | 2.00 |  |
| **HV > MD – 3 second HRF lag** | |  |  |  |  |  |
| *No differences* | |  |  |  |  |  |
| **HV – 5 second HRF lag** | |  |  |  |  |  |
| 1 | Left Rolandic operculum | -44 | -26 | 16 | 2.84 | 174096 |
|  | Left supplementary motor area | -2 | -10 | 52 | 2.66 |  |
|  | Left superior temporal gyrus | -50 | -14 | 6 | 2.64 |  |
|  | Left putamen | -32 | -6 | -4 | 2.57 |  |
| 2 | Right postcentral gyrus | 32 | -24 | 48 | 2.34 | 21376 |
|  | Right Rolandic operculum | 48 | 0 | 12 | 2.31 |  |
|  | Right precentral gyrus | 36 | -14 | 50 | 2.27 |  |
|  | Right insula | 42 | 0 | 0 | 2.22 |  |
| **MD – 5 second HRF lag** | |  |  |  |  |  |
| 1 | Right cerebellum 6 | 20 | -52 | -22 | 3.11 | 68408 |
|  | Vermis 4+5 | 4 | -54 | -6 | 3.09 |  |
|  | Right cerebellum 4+5 | 26 | -36 | -28 | 3.07 |  |
|  | Right cerebellum 4+5 | 14 | -48 | -20 | 3.06 |  |
| 2 | Left supplementary motor area | -4 | -20 | 52 | 3.04 | 224080 |
|  | Left middle cingulate gyrus | -2 | -8 | 46 | 3.03 |  |
|  | Left precentral gyrus | -32 | -12 | 60 | 2.91 |  |
|  | Left middle cingulate gyrus | -20 | -28 | 46 | 2.88 |  |
| 3 | Left middle temporal gyrus | -36 | -58 | 0 | 2.31 | 5512 |
|  | Left fusiform gyrus | -36 | -48 | -6 | 2.31 |  |
|  | Left fusiform gyrus | -38 | -46 | -16 | 2.15 |  |
|  | Left fusiform gyrus | -32 | -36 | -24 | 1.91 |  |
| **HV > MD – 5 second HRF lag** | |  |  |  |  |  |
| 1 | Left precentral gyrus | -32 | -8 | 62 | -2.50 | 2248 |
| 2 | Left postcentral gyrus | -32 | -34 | 46 | -2.17 | 232 |
| **HV – 7 second HRF lag** |  |  |  |  |  |  |
| 1 | Left middle cingulate gyrus | 0 | -6 | 50 | 2.94 | 260928 |
|  | Left middle cingulate gyrus | -2 | -14 | 48 | 2.92 |  |
|  | Left insula | -36 | -8 | 2 | 2.82 |  |
|  | Right fusiform gyrus | 32 | -46 | -16 | 2.78 |  |
| 2 | Right Rolandic operculum | 48 | -2 | 10 | 2.84 | 55896 |
|  | Right Rolandic operculum | 44 | -28 | 20 | 2.63 |  |
|  | Right insula | 38 | -16 | 14 | 2.38 |  |
|  | Right postcentral gyrus | 44 | -18 | 44 | 2.32 |  |
| **MD – 7 second HRF lag** |  |  |  |  |  |  |
| 1 | Left middle cingulate gyrus | -20 | -32 | 46 | 3.37 | 270256 |
|  | Left supplementary motor area | 0 | 6 | 56 | 3.31 |  |
|  | Right middle cingulate gyrus | 2 | -16 | 48 | 3.31 |  |
|  | Left supramarginal gyrus | -52 | -22 | 34 | 3.18 |  |
| 2 | Right fusiform gyrus | 26 | -50 | -12 | 3.17 | 81280 |
|  | Right cerebellum 6 | 14 | -68 | -12 | 3.16 |  |
|  | Right fusiform gyrus | 26 | -36 | -18 | 3.14 |  |
|  | Vermis 4+5 | 4 | -46 | -8 | 3.09 |  |
| 3 | Left fusiform gyrus | -38 | -50 | -10 | 2.20 | 2488 |
|  | Left lingual gyrus | -34 | -56 | 0 | 1.92 |  |
| **HV > MD – 7 second HRF lag** | |  |  |  |  |  |
| 1 | Right putamen | 32 | -6 | 2 | 2.30 | 1136 |
|  | Right insula | 40 | -8 | -6 | 2.21 |  |
|  |  |  |  |  |  |  |
| 1 | Left precentral gyrus | -34 | -2 | 60 | -2.67 | 9000 |
|  | Right supplementary motor area | 8 | -6 | 70 | -2.35 |  |
|  | Left supplementary motor area | -4 | 4 | 62 | -2.24 |  |
| 2 | Right precentral gyrus | 46 | 6 | 48 | -2.30 | 664 |
| 3 | Right postcentral gyrus | 50 | -22 | 46 | -2.19 | 104 |
| 4 | Left postcentral gyrus | -36 | -34 | 48 | -2.13 | 152 |
| **HV – 9 second HRF lag** | |  |  |  |  |  |
| 1 | Right cerebellum 4+5 | 20 | -48 | -22 | 3.16 | 355808 |
|  | Right Rolandic operculum | 46 | 2 | 8 | 3.12 |  |
|  | Right lingual gyrus | 12 | -52 | -6 | 3.06 |  |
|  | Right lingual gyrus | 12 | -66 | -10 | 3.01 |  |
| **MD – 9 second HRF lag** | |  |  |  |  |  |
| 1 | Vermis 6 | -2 | -60 | -16 | 3.10 | 85552 |
|  | Right cerebellum 6 | 26 | -60 | -22 | 3.00 |  |
|  | Right cerebellum 4+5 | 26 | -32 | -24 | 2.96 |  |
|  | Right fusiform gyrus | 24 | -56 | -14 | 2.95 |  |
| 2 | Right supplementary motor area | 4 | -18 | 54 | 3.09 | 179368 |
|  | Left Rolandic operculum | -46 | -20 | 12 | 2.97 |  |
|  | Left precentral gyrus | -30 | -20 | 50 | 2.97 |  |
|  | Left supplementary motor area | -4 | 0 | 68 | 2.94 |  |
| 3 | Right precentral gyrus | 50 | 0 | 46 | 2.83 | 55144 |
|  | Right precentral gyrus | 46 | -14 | 50 | 2.78 |  |
|  | Right Rolandic operculum | 58 | -2 | 10 | 2.76 |  |
|  | Right insula | 50 | 10 | -2 | 2.54 |  |
| **HV > MD – 9 second HRF lag** | |  |  |  |  |  |
| 1 | Right putamen | 32 | -8 | 2 | 2.32 | 736 |
| 2 | Left thalamus | -22 | -26 | 0 | 2.16 | 80 |
|  |  |  |  |  |  |  |
| 1 | Right middle frontal gyrus | 44 | 0 | 54 | -2.48 | 3408 |
| 2 | Right supplementary motor area | 4 | 0 | 70 | -2.33 | 2728 |
|  | Right supplementary motor area | 10 | -14 | 68 | -2.18 |  |
| 3 | Paracentral lobule | 0 | -32 | 60 | -2.16 | 136 |


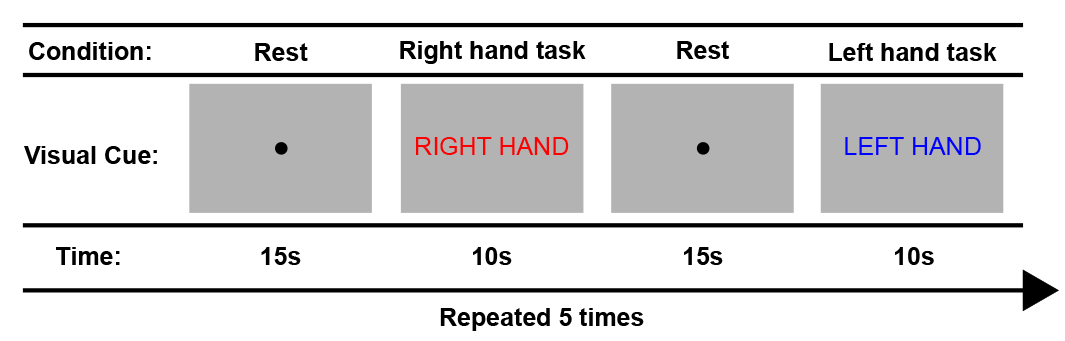


**Supplementary Fig 1.** Schematic overview of the fMRI finger tapping task paradigm.


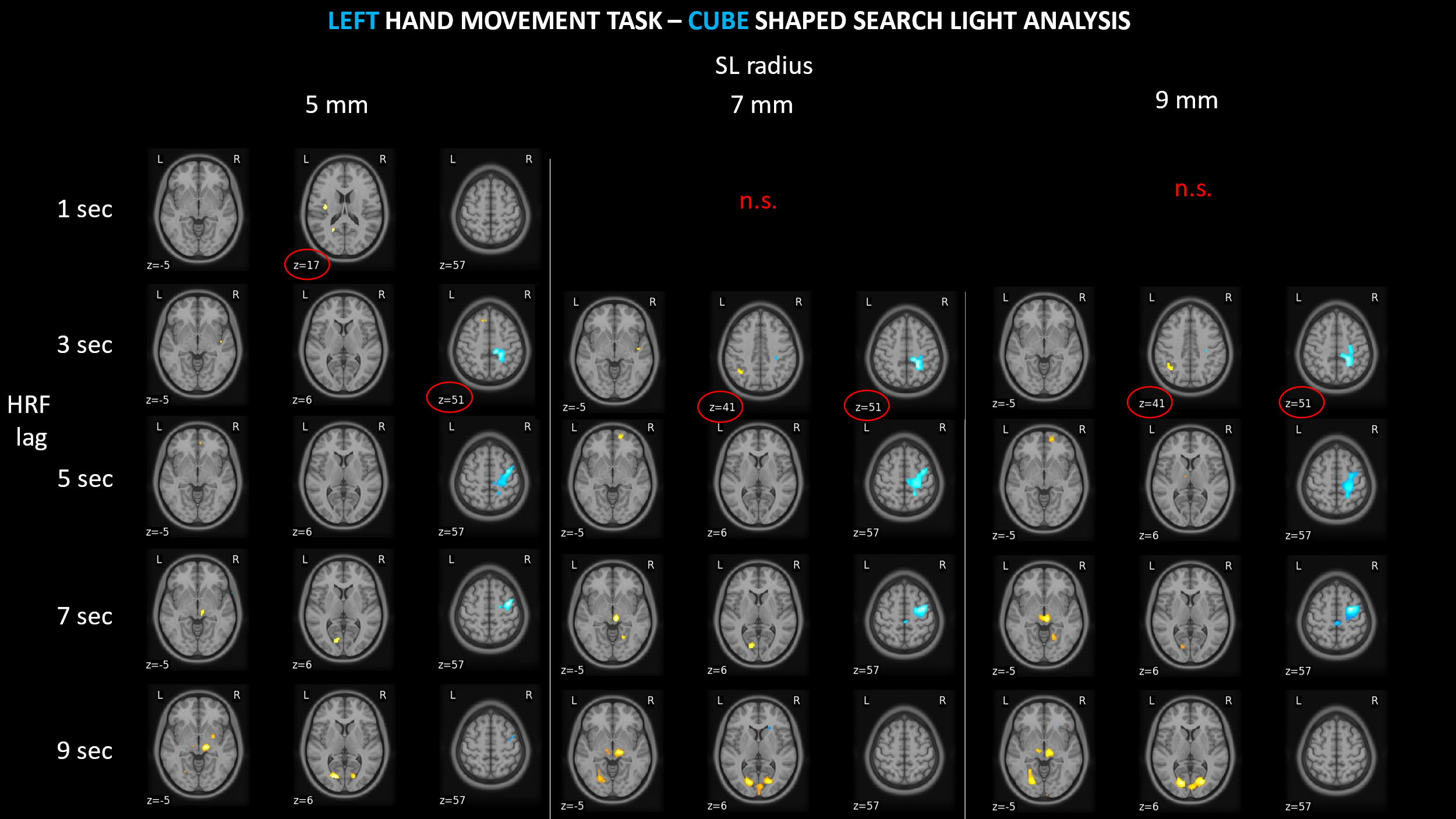


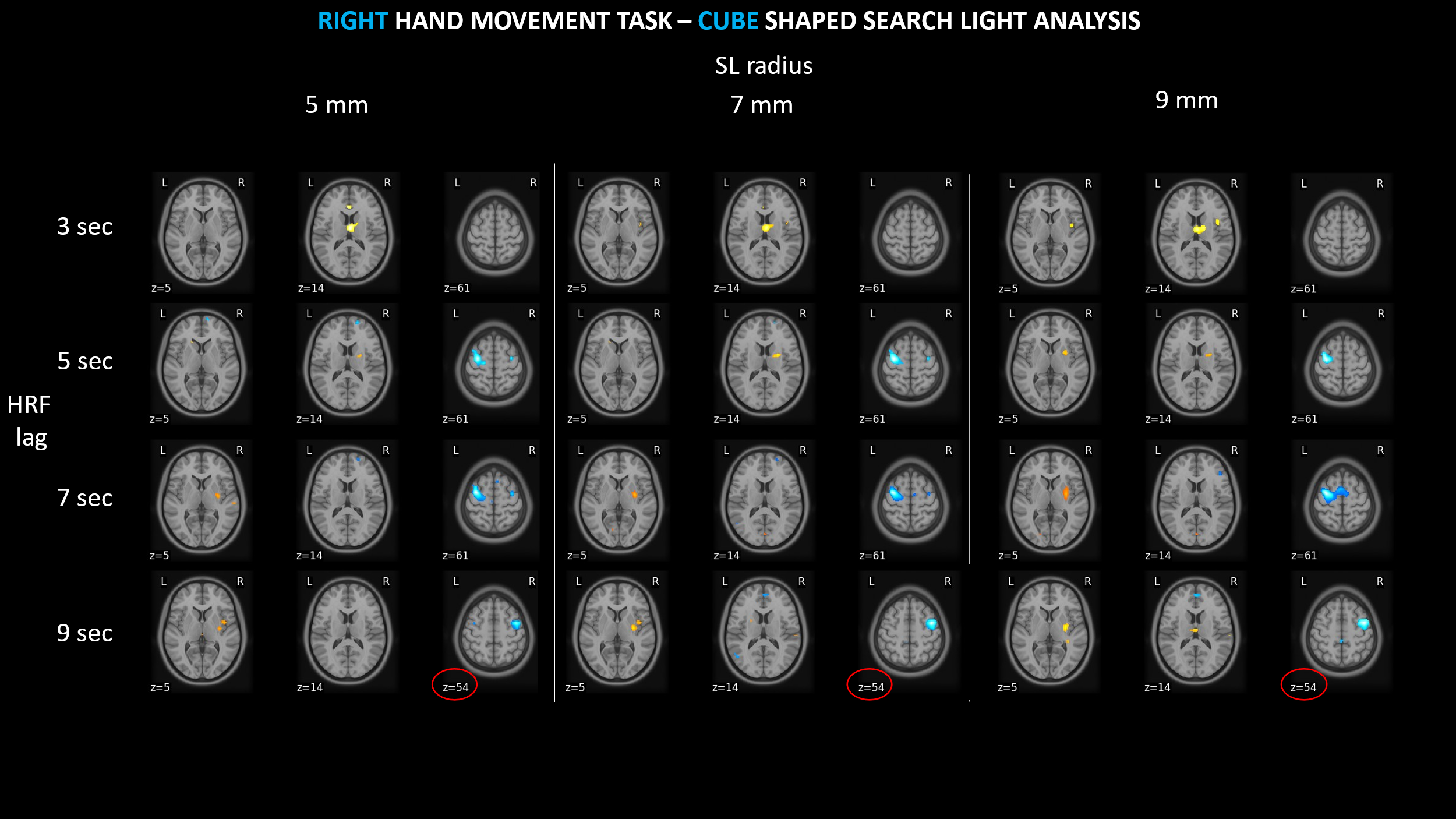


**Supplementary Fig 2.** results of various searchlight radii on classification accuracy and cluster localization for the left and right hand tapping tasks.
